# Quantitative analysis of smokeless powder particles in post‐blast debris via gas chromatography/vacuum ultraviolet spectroscopy (GC/VUV)

**DOI:** 10.1111/1556-4029.15037

**Published:** 2022-04-03

**Authors:** Madison Reavis, John Goodpaster

**Affiliations:** ^1^ Forensic and Investigative Sciences Department Indiana University—Purdue University Indianapolis Indianapolis Indiana USA

**Keywords:** forensic explosive analysis, gas chromatography, GC, GC/VUV, improvised explosive device, post‐blast debris, smokeless powder, vacuum ultraviolet spectroscopy, VUV

## Abstract

Forensic analysis of smokeless powder particles recovered from the debris of an improvised explosive device can provide information about the type of smokeless powder used and can aid investigation efforts. In this study, quantitative methods were used to yield information about the difference in the chemical composition of the particles pre‐ and post‐blast. The technique, gas chromatography/vacuum ultraviolet spectroscopy (GC/VUV), was able to quantify nitroglycerin, 2,4‐dinitrotoluene, diphenylamine, ethyl centralite, and di‐n‐butyl phthalate in pre‐ and post‐blast smokeless powder particles using heptadecane as an internal standard. Post‐blast debris was obtained via controlled explosions with assistance from the Indiana State Police Bomb Squad. Two galvanized steel and two polyvinyl chloride pipe bombs were assembled. Two devices contained single‐base smokeless powder and two contained double‐base smokeless powder. 2,4‐dinitrotoluene and diphenylamine were successfully quantified in the single‐base smokeless powder post‐blast debris while nitroglycerin, diphenylamine, and ethyl centralite were successfully quantified in the double‐base smokeless powder post‐blast debris. Compounds were detected at concentrations as low as 9 μg of 2,4‐dinitrotoluene per mg, <3 μg of diphenylamine per mg, 131 μg of nitroglycerin per mg, and <3 μg of ethyl centralite per mg. Concentration changes between pre‐ and post‐blast smokeless powder particles were determined as well as microscopic differences between pre‐ and post‐blast debris for both smokeless powders in all devices. To our knowledge, this is the first use of GC/VUV for the quantification of explosives.

**Highlights**

- GC/VUV successfully quantified organic components in post‐blast smokeless powder particles.
- GC/VUV can be used for quantification using an internal standard.
- The chemical composition of smokeless powder significantly changes from pre‐ to post‐blast.
- There are significant microscopic changes in smokeless powder particles from pre‐ to post‐blast.

## 1 INTRODUCTION

Bombings in the United States are on the rise for the first time since 2016, with a 71% increase in bombing incidents from 2019 to 2020 [1]. Of the 428 bombings in the United States in 2020, Improvised Explosive Devices (IEDs) were utilized in 177, and of the known main charges used, commercial explosive propellants were one of the three most common [1]. The most prominent type of commercial explosive propellants used in IEDs is smokeless powders (SPs) [2]. Smokeless powders are low explosives that are readily available to purchase for use as reloading powder [3, 4], but when used as the filler in IEDs they can cause extensive damage [5].

Smokeless powders are classified based on their chemical composition. Nitrocellulose (NC) is the energetic material in single‐base SP, NC and nitroglycerin (NG) in double‐base SP, and NC, NG, and nitroguanidine in triple‐base SP [6]. In addition to the energetic material, several organic compounds are added to the powder as stabilizers, plasticizers, flash suppressants, deterrents, opacifiers, and dyes [7]. In the analysis of post‐blast debris, the presence of these compounds indicates that an SP was likely used [8], and from this, it may be possible to identify the explosive manufacturer [9]. The collection and subsequent analysis of post‐blast debris can not only lead to explosive identification, but the data produced can potentially be used as evidence in a court of law or to aid investigation efforts [10, 11].

Smokeless powders have been widely studied using a variety of chemical and instrumental techniques. In general, the most common instruments used to analyze SPs and their components are liquid and gas chromatography. Liquid chromatography has been coupled with QTOF‐MS [12], MS/MS [13], DAD [8], and UV–Vis spectrometry [2] for the qualitative detection of intact, unburned SP samples. Gradient reversed‐phase LC‐ESIMS has been used for the quantification of organic additives differentiate between SPs [14]. HS‐SPME has been paired with GC/FID [15], GC/MS [4], and GC‐micro‐ECD for the identification and quantification of organic additives in intact, unburned SP samples [4]. Total Vaporization‐SPME (TV‐SPME) GC/MS has been used for the quantification of SP residues in real post‐blast debris samples from both PVC and steel IEDs [16, 17]. Cryofocusing capillary microextraction has been paired with GC/MS as a HS extraction device for qualitative analysis of intact SPs [18]. Other techniques like HS‐SPME IMS [4], online SPE‐QqQ‐MS [19], FTIR‐photoacoustic detection [3], CE [9], and TLC [9] have also been used to analyze intact SPs.

Although mass spectrometry is a powerful technique, it has difficulty differentiating isomeric and isobaric compounds and complex software must be used to deconvolve co‐eluting compounds with similar fragmentation patterns [20, 21]. Vacuum ultraviolet spectroscopy is a universal detection system that has been shown to have better specificity [22] and selectivity than MS [20] for some analytes, including nitrate ester explosives, drugs, and petroleum compounds. The VUV detector simultaneously scans wavelengths of 125–430 nm, which range from the VUV to the UV region. Nearly all molecules absorb in this wavelength range due to $n\to \sigma^{*}$, $\sigma \to \sigma^{*}$, $\pi \to \pi^{*}$, and $n\to \pi^{*}$ electronic transitions [21]. VUV spectra are specific to each analyte, making this tool useful to observe the isomeric, isobaric, and co‐eluting compounds that are not easily identified by MS. Since its availability as a benchtop spectrometer, GC/VUV has been used for a variety of applications including fuels [23, 24, 25, 26], food/fragrance products [27, 28, 29], environmental [30, 31, 32], and forensic samples [22, 33, 34, 35, 36, 37, 38, 39, 40, 41, 42, 43, 44, 45, 46, 47, 48].

GC/VUV has been used to study the thermal degradation of nitrate ester and nitramine explosives [22, 44, 46, 47] and an optimized method for explosives analysis with application to post‐blast debris has been reported [45]. Cruse et al have also identified SP organic additives in both unburned SP [22] and real post‐blast debris [45] and added the subsequent spectra to the VUV spectral library. The compounds identified were nitroglycerin, diphenylamine, ethyl centralite, and di‐n‐butyl phthalate.

In contrast to previous studies, this study will focus on the quantitative analysis of SP. Although the legal question in explosives investigations is which explosive was used (not how much), the quantitation of the components of SPs will allow limits of detection (LODs) to be calculated and determine the sensitivity required of the GC/VUV to detect these compounds [16]. The analysis of SP particles in post‐blast debris can also yield information about the difference in chemical composition of the particle pre‐ and post‐blast, and how this differs based on the container used.

This work quantifies nitroglycerin, 2,4‐dinitrotoluene (2,4‐DNT), diphenylamine (DPA), ethyl centralite (EC), and di‐n‐butyl phthalate in pre‐ and post‐blast SP particles using heptadecane as an internal standard. To our knowledge, this is the first use of GC/VUV for the quantification of explosives.

## 2 MATERIALS & METHODS

### 2.1 Chemicals

Nitroglycerin (1000 μg/ml in methanol) was purchased from Restek. Diphenylamine and n‐heptadecane were purchased from Acros Organics. Dinitrotoluene was purchased from Spectrum Chemicals. Ethyl centralite was purchased from Aldrich Chemistry. Di‐n‐butyl phthalate was purchased from Supelco. Acetone was purchased from Fisher Chemical. Alliant Red Dot and IMR 4064 were purchased locally. IMR 4064 is a single‐base rod‐shaped smokeless powder designed to burn from the inside out. Alliant Red Dot is a double‐base disc‐shaped smokeless powder with red identifiers. The chemical composition of the red identifiers in Alliant Red Dot is the same as the other particles present, only with a colorant added.

### 2.2 Post‐blast debris generation and collection

Post‐blast debris was obtained via controlled explosions with assistance from the Indiana State Police Bomb Squad. Two galvanized steel and two polyvinyl chloride (PVC) IEDs were assembled. For both materials, one device contained IMR 4064 and the other contained Alliant Red Dot. Each device was placed in a vented 2′ × 2′ × 2′ steel box to help retain the post‐blast debris for collection.

### 2.3 Sample preparation

Acetone was chosen as the solvent for this study so that it would effectively dissolve any nitrocellulose present and thus effectively disrupt the matrix of the sample. An internal standard solution of 100 ppm heptadecane in acetone was made and used as the solvent for all experiments. A mixture of nitroglycerin, 2,4‐dinitrotoluene, diphenylamine, ethyl centralite, and dibutyl phthalate in internal standard solution was made. First, the nitroglycerin in methanol (1000 μg/ml) was concentrated using a nitrogen blow down apparatus and reconstituted in internal standard solution. After this step, each of the remaining components were added to the mixture at the same concentration as nitroglycerin. Using this mixture, calibrants were made for subsequent analysis by GC/VUV. Three calibration curves were used to quantify the data discussed in this paper. All curves included concentrations of 3, 5, 10, 30, 50, and 100 ppm. The curves used to quantify Alliant Red Dot in the steel device, IMR 4064 in both the steel and PVC devices, and standard IMR 4064 included higher concentrations of 223, 446, and 892 ppm while the curve used to quantify standard Alliant Red Dot and Red Dot in the PVC devices included high concentrations of 280, 559, and 1118 ppm.

Individual solutions of Alliant Red Dot and IMR 4064 were prepared at 1000 ppm of each smokeless powder in internal standard solution. Before injection into the GC/VUV, each solution was filtered with a PTFE filter to ensure no particulates were injected into the GC/VUV.

Intact particles were recovered from the post‐blast debris using forceps while observing the debris under an Olympus SZ61 stereomicroscope equipped with a Fiber‐Lite MI‐150 High Intensity Illuminator from Dolan‐Jenner Industries. Solutions containing the post‐blast particles were prepared at 1000 ppm in internal standard solution. These solutions were also filtered using a PTFE filter to ensure no particulates were injected into the GC. Calibrants were prepared and analyzed on the same day as the post‐blast samples to ensure reproducible and reliable quantification.

### 2.4 Gas chromatography

1 μl of each sample was injected into an Agilent 7890B gas chromatograph equipped with an Agilent 7693 autosampler and a multimode inlet. The sample was introduced using hydrogen carrier gas at 2.4 ml/min. The multimode inlet was in splitless mode with a temperature program beginning at 50°C and ramped to 200°C at 900°C/min. The analytes were then separated by a 15 m × 320 μm × 0.25 μm Restek Rtx®‐5MS column. The oven temperature began at 50°C for 30 s, and was then ramped at 20°C/min to 250°C. The same oven temperature program was used for all samples. The GC and VUV parameters used here were previously optimized [45].

### 2.5 Vacuum ultraviolet spectroscopy

After separation in the GC, the analytes were introduced into a VUV Analytics VGA‐101 VUV spectrometer at a transfer line and flow cell temperature of 300°C. Samples were analyzed using a scan rate of 4.5 Hz, a deuterium lamp at 1.2 V, a make‐up gas pressure of 0.00 psi, and a spectral range of 120–430 nm [45]. To improve the signal‐to‐noise ratio, the resulting chromatograms were analyzed using absorbance data from 125–240 nm.

### 2.6 Calculations

Signal‐to‐noise ratios were calculated using a wavelength filter from 125–240 nm. Peak height was used to define signal. The standard deviation of the 20 baseline points preceding the peak was defined as noise. Nine calibration concentrations ranging from 3–1118 ppm were used for calculations. Limits of detection were calculated by plotting log(S/N) versus log(concentration) and determining the concentration of analyte that yielded a S/N of 3.

## 3 RESULTS & DISCUSSION

### 3.1 GC/VUV calibration for quantification

Three sets of calibrants were used for quantification because the debris was analyzed on three separate days. The linear ranges, limits of detection, and mass on column reported in Table 1 are the broadest range, lowest concentration, and smallest mass on column detected out of all three calibration sets. *R* ^2^ values for each calibration were typically greater than 0.99.

**TABLE 1:** The broadest linear range, lowest limit of detection (LOD), and mass on column found for each compound from three sets of calibrants is shown above

	Nitroglycerin	2,4‐Dinitrotoluene	Diphenylamine	Ethyl Centralite	Di‐n‐butyl phthalate
Linear Range (ppm)	30–892	3–1118	3–446	3–892	10–1118
LOD [ng/μl]	1.90	0.256	0.114	0.0802	0.352
Mass on Column (ng)	1.90	0.256	0.114	0.0802	0.352

**FIGURE 1:**
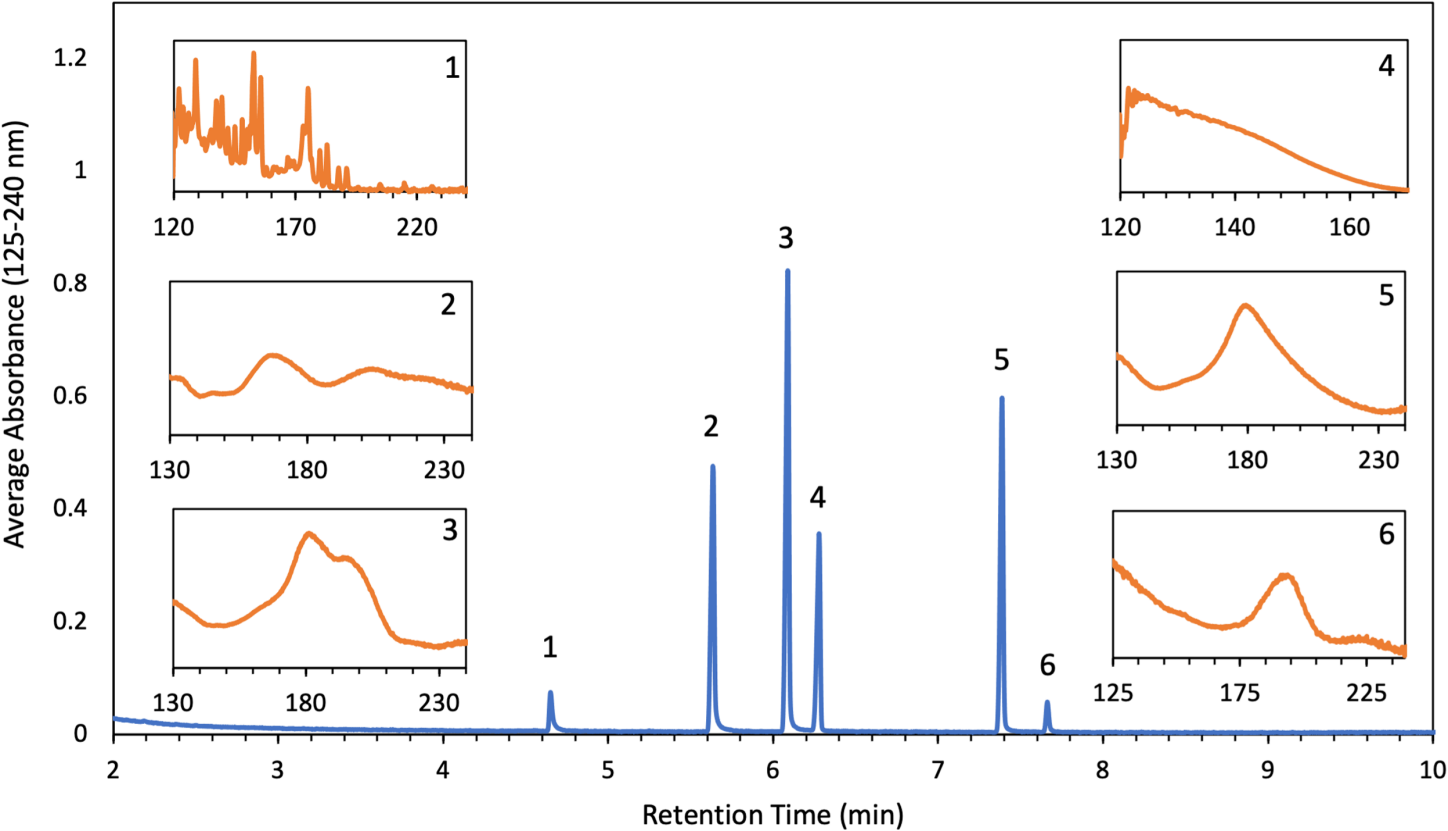
GC/VUV calibrant chromatogram with corresponding VUV spectra for nitroglycerin (1), 2,4‐dinitrotoluene (2), diphenylamine (3), heptadecane (4), ethyl centralite (5), and di‐n‐butyl phthalate (6)

Each calibrant contained components from IMR 4064, Alliant Red Dot, and an internal standard. The retention times for nitroglycerin, 2,4‐dinitrotoluene, diphenylamine, ethyl centralite, and di‐n‐butyl phthalate were 4.66 min, 5.64 min, 6.09 min, 7.39 min, and 7.67 min, respectively. The retention time for heptadecane was 6.28 min. Heptadecane was selected as the internal standard because odd numbered alkanes are not found in smokeless powders, and its retention time was similar to the other analytes in solution. Figure 1 is an example chromatogram with corresponding VUV spectra. All chromatograms were generated by averaging absorbance values from 125–240 nm to improve S/N.

Something to note in Figure 1 is the VUV spectrum of nitroglycerin (box 1). Unlike the other VUV spectra included, this spectrum is rich in spectral details and contains narrow absorption bands. These spectral details are a result of the thermal degradation of nitroglycerin in the VUV flow cell into a mixture of nitric oxide, carbon monoxide, formaldehyde, oxygen, and water [22]. The nitroglycerin spectrum produced is a combination of the VUV spectra of each of the decomposition products. The complete thermal degradation of nitroglycerin occurs at a flow cell temperature of 280°C. At temperatures below this, the molecule is still intact that results in a broad VUV spectrum consistent with the VUV spectrum of water and oxygen [22].

### 3.2 Microscopic examination of pre‐ and post‐blast particles

#### 3.2.1 IMR 4064

**FIGURE 2:**
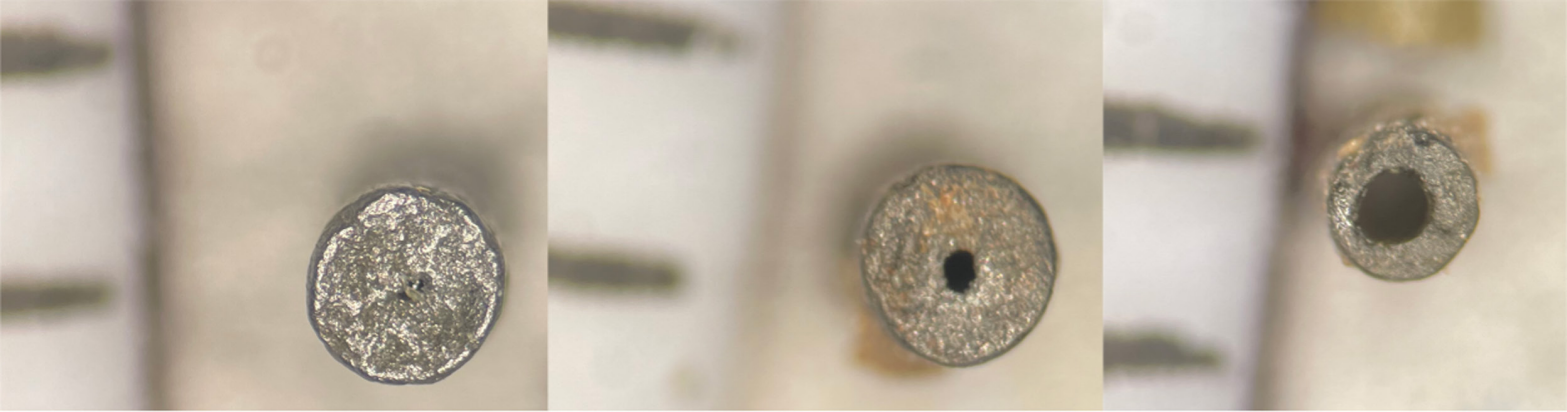
Photographs of IMR 4064 particles pre‐blast (left), recovered from a PVC device (middle), and recovered from a steel device (right) with 1 mm scale

IMR 4064 is a single‐base rod‐shaped smokeless powder designed to burn from the inside out. This phenomenon is illustrated in Figure 2. When both pre‐ and post‐blast particles were viewed vertically, a small opening was seen in the center of the particle. Both PVC and steel post‐blast particles were smaller in overall size, but the size of this opening increased more in the steel particles than the PVC particles. This is likely because the steel device reached a higher temperature before failing, so more of the particle was consumed in the explosion.

#### 3.2.2 Alliant red dot

**FIGURE 3:**
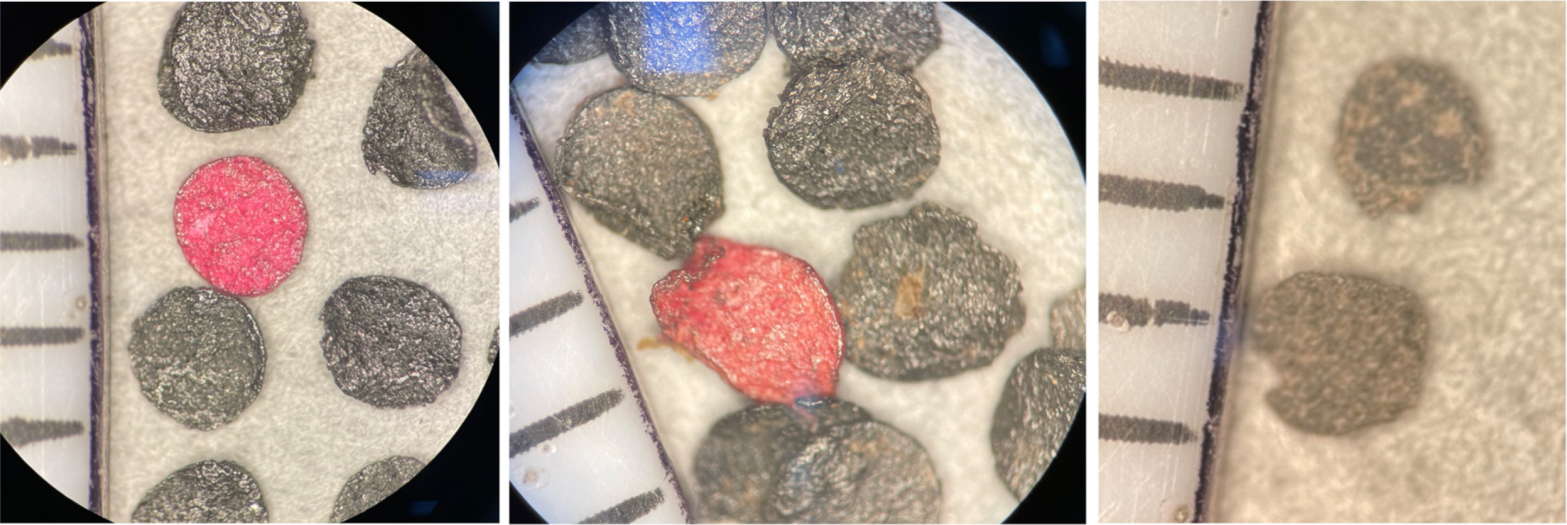
Photographs of Alliant red dot particles pre‐blast (left), recovered from a PVC device (middle), and recovered from a steel device (right) with 1 mm scale. Steel particles are from a controlled explosion in 2018

Alliant Red Dot is a double‐base disc‐shaped smokeless powder with red identifiers. If red particles are found in post‐blast debris, it is a clear indication that Red Dot was used. The particles recovered from both the PVC and steel devices appear to be partially burned and have less of a circular shape than the pre‐blast particles (Figure 3).

### 3.3 Quantification of pre‐ and post‐blast particles via GC/VUV

Due to the nature of these of experiments, only one device of each type was analyzed (e.g., one steel IED filled with IMR 4064), so the SP replicates are particles from the same device. The conclusions made about concentration changes in the particles from pre‐ to post‐blast would be strengthened if more devices of the same type were utilized. This would allow SP particles to be compared between devices to ensure the concentration changes observed from pre‐ to post‐blast particles are due to container type.

#### 3.3.1 IMR 4064

**FIGURE 4:**
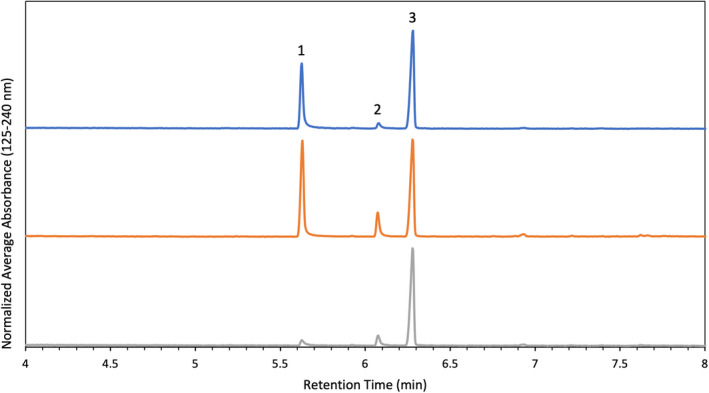
Chromatograms of IMR 4064 particles pre‐blast (top), recovered from a PVC device (middle), and recovered from a steel device (bottom). The peaks present correspond to 2,4‐dinitrotoluene (1), diphenylamine (2), and heptadecane (3)

The components of interest in IMR 4064 were 2,4‐DNT and DPA, both of which were detected and quantified in all IMR 4064 samples. Figure 4 shows stacked chromatograms of IMR 4064 particles pre‐blast (top), recovered from a PVC device (middle), and recovered from a steel device (bottom). There was approximately a 5‐fold decrease in concentration of 2,4‐DNT in the steel post‐blast particles compared to both the pre‐blast and PVC post‐blast particles (Table 2). This change in concentration was found to be statistically significant. The decrease in 2,4‐DNT in the particles collected from the steel device is likely because the steel device reached higher temperatures before failure, so the compound was consumed in the reaction. Although not statistically significant, there was a relative increase in concentration of 2,4‐DNT and DPA from the pre‐blast particles to PVC post‐blast particles. This is likely because the size of the particle decreased, but neither the 2,4‐DNT or DPA were consumed in the reaction so there was a relative increase in their concentration. The relative increase in concentration of DPA in the particles collected from the steel device is likely because of this same reasoning.

**TABLE 2:** This table shows the average concentration of 2,4‐dinitrotoluene and diphenylamine found in standard and post‐blast single‐base smokeless powder particles (IMR 4064). Concentration is reported in μg compound/mg of powder. These values are the average of three replicates

Device	2,4‐Dinitrotoluene		Diphenylamine	
	Average concentration	Standard deviation	Average concentration	Standard deviation
Standard IMR 4064	48	8.7	3.9	2.9
PVC IMR 4064	57	6.4	7.6	1.1
Steel IMR 4064	9.3	1.8	4.3	2.1

#### 3.3.2 Alliant red dot

**FIGURE 5:**
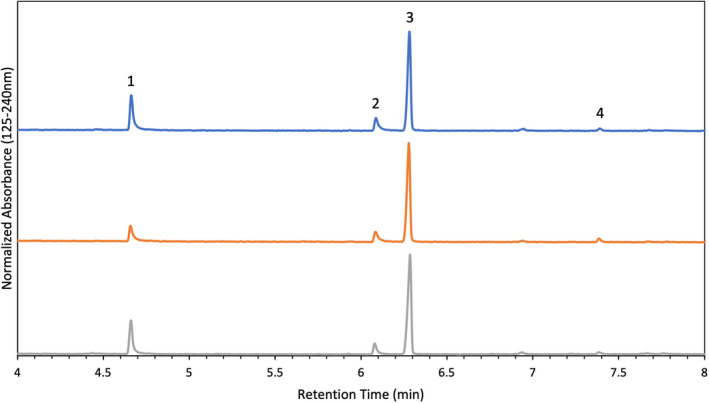
Chromatograms of Alliant red dot particles pre‐blast (top), recovered from a PVC device (middle), and recovered from a steel device (bottom). The peaks present correspond to nitroglycerin (1), diphenylamine (2), heptadecane (3), and ethyl centralite (4)

The components of interest in Red Dot were nitroglycerin, diphenylamine, ethyl centralite, and di‐n‐butyl phthalate. Nitroglycerin, diphenylamine, and ethyl centralite were detected and quantified in all Red Dot samples. Figure 5 shows stacked chromatograms of Alliant Red Dot particles pre‐blast (top), recovered from a PVC device (middle), and recovered from a steel device (bottom). The average concentration of components (μg compound/mg of powder) found in pre‐ and post‐blast intact particles is reported in Table 3. There were a limited number of intact particles from the steel device, and because of this only one run was able to be performed (no standard deviation). There was approximately a 1.5‐fold decrease in nitroglycerin concentration from the pre‐blast particles to both the PVC and steel post‐blast particles, a statistically significant change in concentration. There was an approximate 2‐fold decrease in DPA and EC concentration in the steel post‐blast particles relative to the pre‐blast and PVC post‐blast particles. These changes in concentration were not found to be statistically significant. The decrease in DPA concentration in the PVC post‐blast particles relative to the pre‐blast particles was found to be statistically significant. The relative increase in EC concentration from the pre‐blast to the PVC post‐blast particles is likely because the size of the particle decreased, but the EC was not consumed in the reaction so there was a relative increase in its concentration.

**TABLE 3:** This table shows the average concentration of nitroglycerin, diphenylamine, and ethyl centralite found in standard and post‐blast double‐base smokeless powder particles (Alliant red dot). Concentration is reported in μg compound/mg of powder. All values are the average of three replicates, except steel values, which were only ran once due to a limited number of intact particles

Device	Nitroglycerin		Diphenylamine		Ethyl Centralite	
	Average concentration	Standard deviation	Average concentration	Standard deviation	Average concentration	Standard deviation
Standard Red Dot	217	7.8	6.9	0.60	1.8	0.53
PVC Red Dot	135	13	5.1	0.76	1.9	0.32
Steel Red Dot	132	‐	2.7	‐	0.58	‐

### 3.4 Principal component analysis of components

**FIGURE 6:**
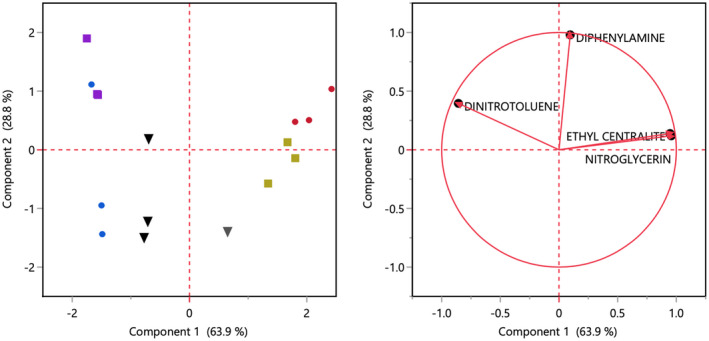
Principal component analysis (PCA) plot and map of IMR 4064 (left side of graph) and red dot (right side of graph) data. Circles correspond to pre‐blast particles, squares correspond to particles recovered from a PVC device, and upside‐down triangles correspond to particles recovered from a steel device

Principal component analysis (PCA) was used to help further visualize how a particle’s chemical composition changes from pre‐ to post‐blast. The PCA plot shown on the left in Figure 6 includes all samples from both the IMR 4064 and Red Dot runs. The data points in quadrants A and B correspond to the IMR 4064 samples while the data points in quadrants C and D correspond to the Red Dot samples. The factors loading plot, shown on the right in Figure 6, is used to identify the change in concentration of a specific component in the PCA plot. For example, when observing the IMR 4064 data in quadrants A and B, the decrease in the concentration of 2,4‐DNT is easy to spot between the pre‐ and post‐blast samples. The same can be said for the decrease in nitroglycerin from pre‐ to post‐blast in the Red Dot samples. This was determined because the factors loading plot indicates that the concentration of 2,4‐DNT decreases from left to right, just as the concentration of nitroglycerin decreases from right to left. PCA was also useful because it illustrates that both the IMR 4064 and Red Dot data are correlated within themselves, but that the two sets of data are not correlated with one another. In the factor loading plot, nitroglycerin and EC are projected on top of each other. This means that the levels of nitroglycerin and EC are highly correlated, while they are both negatively correlated with 2,4‐DNT.

## 4 CONCLUSION

GC/VUV has been used to successfully quantify nitroglycerin, 2,4‐dinitrotoluene, diphenylamine, ethyl centralite, and di‐n‐butyl phthalate using heptadecane as an internal standard. Several statistically significant concentration changes between pre‐ and post‐blast smokeless powder particles were determined. Microscopic differences were observed between pre‐ and post‐blast debris for both IMR 4064 and Alliant Red Dot in both PVC and steel IEDs. Future work includes creating a separate calibration curve for nitroglycerin to help improve LOD calculations and analyzing multiple IEDs of the same type to compare smokeless powder particles between devices to see if the concentration changes of the organic components are solely due to container type.
